# First crystal structures of 1-deoxy-d-xylulose 5-phosphate synthase (DXPS) from *Mycobacterium tuberculosis* indicate a distinct mechanism of intermediate stabilization

**DOI:** 10.1038/s41598-022-11205-9

**Published:** 2022-05-04

**Authors:** Robin M. Gierse, Rick Oerlemans, Eswar R. Reddem, Victor O. Gawriljuk, Alaa Alhayek, Dominik Baitinger, Harald Jakobi, Bernd Laber, Gudrun Lange, Anna K. H. Hirsch, Matthew R. Groves

**Affiliations:** 1 Helmholtz Institute for Pharmaceutical Research Saarland (HIPS)-Helmholtz Centre for Infection Research (HZI), Campus Building E 8.1, 66123 Saarbrücken, Germany; 2 Department of Pharmacy, Saarland University, Campus Building E8.1, 66123 Saarbrücken, Germany; 3 Stratingh Institute for Chemistry, University of Groningen, Nijenborgh 7, 9747 AG Groningen, The Netherlands; 4 Department of Drug Design, Groningen Research Institute of Pharmacy, University of Groningen, Antonius Deusinglaan 1, 9700 AV Groningen, The Netherlands; 5 São Carlos Institute of Physics, University of São Paulo, Av. João Dagnone, 1100-Santa Angelina, São Carlos, SP 13563-120 Brazil; 6 Research & Development Crop Science, Bayer AG, Industriepark Höchst, 65926 Frankfurt, Germany

**Keywords:** Enzymes, X-ray crystallography, Structural biology, Molecular modelling, X-ray crystallography, Enzyme mechanisms

## Abstract

The development of drug resistance by *Mycobacterium tuberculosis* and other pathogenic bacteria emphasizes the need for new antibiotics. Unlike animals, most bacteria synthesize isoprenoid precursors through the MEP pathway. 1-Deoxy-d-xylulose 5-phosphate synthase (DXPS) catalyzes the first reaction of the MEP pathway and is an attractive target for the development of new antibiotics. We report here the successful use of a loop truncation to crystallize and solve the first DXPS structures of a pathogen, namely *M. tuberculosis* (*Mt*DXPS). The main difference found to other DXPS structures is in the active site where a highly coordinated water was found, showing a new mechanism for the enamine-intermediate stabilization. Unlike other DXPS structures, a “fork-like” motif could be identified in the enamine structure, using a different residue for the interaction with the cofactor, potentially leading to a decrease in the stability of the intermediate. In addition, electron density suggesting a phosphate group could be found close to the active site, provides new evidence for the D-GAP binding site. These results provide the opportunity to improve or develop new inhibitors specific for *Mt*DXPS through structure-based drug design.

## Introduction

The drug resistance of *Mycobacterium tuberculosis*, the causative agent for tuberculosis (TB), is a growing problem. Diagnosis and treatment of TB are both challenging tasks, the latter lasting for 6 months or more^1^. Although the WHO declared TB a global public health emergency in 1993, difficult and often incomplete treatment led to the emergence of resistant strains^2,3^. Over the years, *M. tuberculosis* has developed mechanisms of resistance against the known first-line “multi-drug resistant TB” and second line “extensively drug-resistant TB” antitubercular agents^4^. This development ultimately led to totally drug resistant TB (TDR-TB), resistant to all known first- and second-line antitubercular agents, observed first in 2008 in Iran and in 2012 in India^5,6^. To treat patients infected with TDR-TB, the development of drugs with new modes of action is urgently needed.

**Figure 1:**
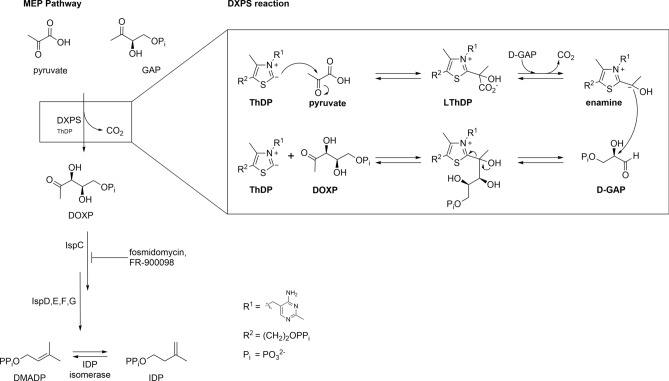
An overview of the MEP pathway and the mechanism of the reaction catalyzed by DXS. Fosmidomycin and its derivative FR-900098 are inhibitors of the DXPS downstream enzyme IspC, validating the pathway as target for the development of new antibiotics.

The 2-*C*-methyl-d-erythritol 4-phosphate (MEP)-pathway offers seven new target enzymes for the development of anti-TB drugs, which should, with a new mode of action, break the resistance of TDR-TB^7,8^. For many bacteria, this pathway is the only source of the terpene building blocks dimethylallyl diphosphate (DMADP) and isopentenyl diphosphate (IDP), essential for the biosynthesis of secondary metabolites (Fig. 1). The IspC inhibitor fosmidomycin is already used in combination therapy for the treatment of malaria, validating this pathway for the development of new drugs^9^. Several projects to develop further antibiotic drugs are currently running, targeting different enzymes of the MEP pathway^10–15^. The homologues of *M. tuberculosis* are also addressed. A series of lipophilic phosphonates that target the IspC enzyme from *E. coli* and *M. tuberculosis* was designed and synthesized. The corresponding co-crystal structure confirms a binding mode similar to that of the natural inhibitor fosmidomycin^16^. Aryl bis-sulfonamides were investigated to inhibit the downstream enzymes of the pathway: the IspF enzyme of *Plasmodium falciparum* and, with slightly lower activity, also the homologue from *M. tuberculosis*^17^. However, to the best of our knowledge, no qualified lead compound targeting MEP-pathway enzymes from *M. tuberculosis* was selected for further development.

In our drug design project, we chose to focus on 1-deoxy-d-xylulose 5-phosphate synthase (DXPS), the first and rate-limiting enzyme of the MEP-pathway^18^. DXPS catalyzes the ThDP-dependent decarboxylation of pyruvate and subsequent carboligation with d-glyceraldehyde-3-phosphate (D-GAP), the second substrate, following a preferred-order, random-sequential reaction mechanism (Fig. 1)^19,20^. Targeting this enzyme has the additional benefit of not only inhibiting the biosynthesis of the terpene precursors, but also the biosynthesis of the vitamins B1 and B6, which are synthesized from the product of DXPS, the branch-point metabolite 1-deoxy-d-xylulose 5-phosphate (DOXP)^21^. A previous ligand-based approach of our group to design ThDP-competitive inhibitors of DXPS, using the *Deinococcus radiodurans* DXPS (drDXPS) crystal structure and an *M. tuberculosis* DXPS (*Mt*DXPS) homology model, resulted in compounds showing activity against *D. radiodurans*, but with significantly less activity against *Mt*DXPS^22^.

The modern drug-design process benefits greatly from structural information of the enzymatic target. The knowledge of the molecular interactions between an inhibitor and its target protein sets the stage for structure-based optimization and therefore holds the potential to speed up the optimization of a hit or lead compound^23,24^. Most enzymes of the MEP pathway are structurally well-characterized, with structures of the enzymes IspC to IspH from multiple species available, both apo and in complex with inhibitors (Table S1), highlighting the interest in structure-based drug design studies for enzymes of this pathway. In contrast, the DXPS enzyme is less explored—with just five protein structures published to date, four from *D.radiodurans* and one from *E. coli*^25–27^. The low number of crystal structures may be a consequence of the enzyme´s susceptibility to proteolytic degradation^25,27,28^ or conformational changes during the catalytic cycle^19,29^—both properties that can make it difficult to obtain a homogeneous protein sample for crystallization. We recently published a modified drDXPS protein with improved crystallographic properties and speculated that our approach should be applicable to all DXPS homologues^27^.

In this report, we describe the application of our truncation strategy on the DXPS homologue of *M. tuberculosis*, as a representative pathogen in the focus of several drug-design projects^30^. Following the truncation approach, we were able to obtain protein crystals and report herein the first pathogenic DXPS structures: a holo structure with bound ThDP and a structure containing the enamine reaction intermediate (Fig. 1). We also docked *Mt*DXPS inhibitors found in the literature and provide a structural rationale for their inhibitory activity. In addition to providing a molecular model of this important anti-infective target, this also shows that our truncation approach is transferable between organisms and has the potential to facilitate the determination of structures from other DXPS homologues in the future.

## Results

### Application of the truncation strategy

In our recent article, we showed that the truncation of a non-conserved loop not visible in the structures of the DXPS enzyme of *D. radiodurans* (drDXPS) improved the crystallization of this target^27^. We hypothesized that this result could also be transferable to other homologues. The DXPS enzyme from *M. tuberculosis* (*Mt*DXPS) was chosen to test this hypothesis. This homologue is of particular interest, as it is from a pathogenic organism and inhibitors targeting the enzyme have been investigated, utilizing a computed homology model due to the lack of an *Mt*DXPS structure^22,40^.

To apply the truncation strategy, we searched the previously published multiple sequence alignment (MSA) for the protein sequence of *M. tuberculosis* and compared it with the sequence of *D. radiodurans*^27^. The corresponding non-conserved loop from *Mt*DXPS has a length of 45 amino acids, comprising amino acids 190–234 (Fig. S1). We replaced this loop with a linker of seven glycines that we visually estimated to be able to compensate for the removed amino acids, according to a homology model obtained for the *Mt*DXPS homologue^22^. The resulting truncated sequence (**SI: Sequences**) of *Mt*DXPS (Δ*Mt*DXPS) was then obtained as a synthetic gene.

### Characterization of the truncated enzyme

The Δ*Mt*DXPS was recombinantly expressed as a soluble protein in good yields of > 50 mg/L, whereas we could express the native protein in a range of 0.5 mg/L in LB-medium. To analyze other effects of the truncation, we compared the wild-type *Mt*DXPS and the truncated Δ*Mt*DXPS, using several biophysical techniques.

Δ*Mt*DXPS integrity was analyzed by LC–MS. The sample showed a single protein with a high purity and a mass of 65,901.8 Da (Fig. S2). This mass is 705 Da lighter than calculated for the full length Δ*Mt*DXPS protein, and the full mass could not be observed. The weight of 705 Da corresponds to the molecular weight of the last six amino acids of the protein, suggesting either incomplete translation, a common issue in protein expression, or degradation of the sample during preparation. The terminal amino acids are often not resolved in protein crystal structures due to their flexibility, so the lack of the last six residues is unlikely to be a concern.

One of the truncation goals was to reduce the degradation of the wild-type *Mt*DXPS enzyme. Over an incubation period of up to 5 days at RT, degradation of Δ*Mt*DXPS could not be detected on SDS-PAGE gels. In contrast, for full length *Mt*DXPS bands corresponding to calculated degradation products of 20 and 40 kDa started to appear after 4 days (Fig. S3). This observation becomes more apparent when the time period is extended to 7 days, where a substantial decrease in band intensity of full length *Mt*DXPS protein can be seen, while Δ*Mt*DXPS is still stable. Therefore, similarly to drDXPS, the loop truncation reduced the tendency for protein degradation, overall increasing the stability. As an additional method to assess protein stability, the melting point (*T*_m_) was determined using a thermal shift assay (TSA). The *T*_m_ obtained for both wild-type and truncated *Mt*DXPS was 45.4 ± 0.4 °C and 52.7 ± 0 °C, respectively. The large difference in melting temperature between the two proteins (∆*T*_m_ = 7.3 °C) strongly suggests that the truncation improved protein stability, which is often beneficial for protein crystallization^31^.

Similarly to drDXPS, the truncation had only small effects on the enzymatic activity of Δ*Mt*DXPS, as shown in Table 1. The Δ*Mt*DXPS enzyme showed a slightly reduced affinity for the substrates and a 2–3 times higher activity, which could originate from a more accessible active site with a smaller degree of conformational variability during the catalytic cycle. Nevertheless, the kinetic parameters are still within the same magnitude and the retained enzymatic activity seems to indicate that no catalytically important residues are impacted by the truncation.

**Table 1:** Comparison of the enzyme kinetics from *Mt*DXPS and Δ*Mt*DXPS.

Enzyme	Pyruvate	D-GAP
*Mt*DXPS 5 µmol/L	*K*_m_: 85 ± 8 µM *k*_cat_: 4.5 ± 0.1 × 10^–3^ s^-1^	*K*_m_: 75 ± 7 µM *k*_cat_: 3.9 ± 0.7 × 10^–3^ s^-1^
Δ*Mt*DXPS 2 µmol/L	*K*_m_: 125 ± 13 µM *k*_cat_: 17.3 ± 0.4 × 10^–3^ s^-1^	*K*_m_: 112 ± 11 µM *k*_cat_: 10.0 ± 0.3 × 10^–3^ s^-1^

### ∆*Mt*DXPS crystal structure

**Figure 2:**
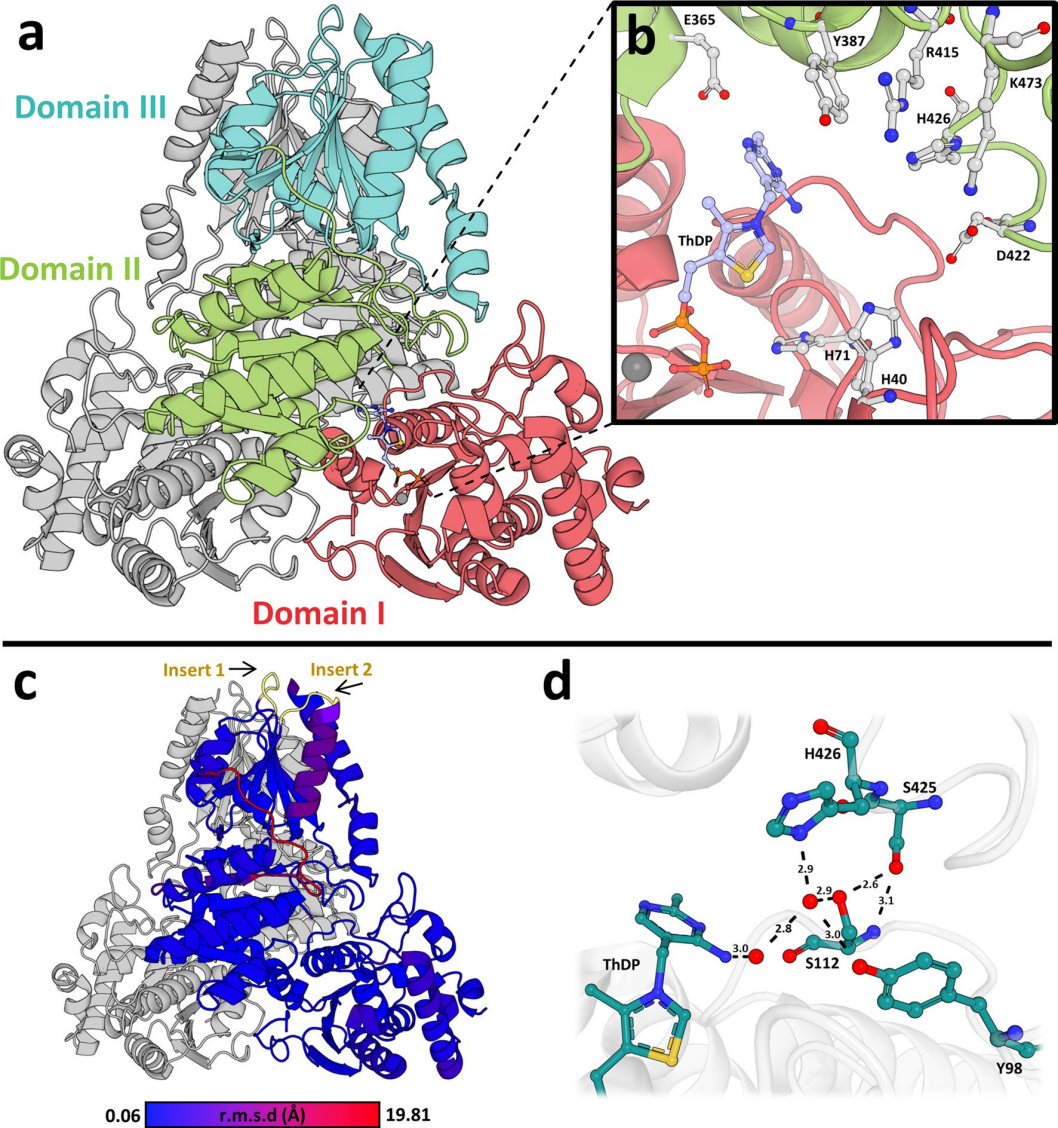
Overview of the holo structure of *ΔMtDXPS*. (**a**) Overview of the Δ*Mt*DXPS structure with highlighted domains. Domain I (res 1–312) in red, domain II (res 313–483) in green and domain III (484–638) in cyan. (**b**) Active site with important residues for catalysis shown in stick representation. (**c**) Structure colored according to C_α_-RMSD to the drDXPS structure (PDB ID: **2O1X**), insertion regions are colored in yellow. (**d**) Hydrogen-bonding network that stabilizes the different H426 conformation and key residues in the network. Water molecules are shown as red spheres and hydrogen bonds as black, dashed lines with distances indicated in Ångström.

We previously performed extensive crystallization screening of the wild-type *Mt*DXPS enzyme, but were unable to obtain protein crystals. In contrast, the Δ*Mt*DXPS protein crystallized within 2 days in several conditions. From the best condition, we were able to determine the crystal structure of the holo protein of Δ*Mt*DXPS to a resolution of 1.85 Å (PDB ID: **7A9H**) (Fig. 2a). The numbering of amino acids discussed in the following text is according to the *Mt*DXPS sequence with the UniProt ID: **P9WNS3** (**SI: Sequences**).

The asymmetric unit of Δ*Mt*DXPS contains a homodimer with each subunit consisting of three distinct domains and a ThDP molecule. Domain I (res 1–312) and Domain II (res 313–483) contribute to the active site, where ThDP is bound. Domain III (res 484–638) makes extensive contacts at the dimer interface (Fig. 2a). Overall, the structure and domain arrangement of *Mt*DXPS show high similarity to the known structures of *E. coli* and *D. radiodurans* DXPS that were described in detail by S. Xiang et al. with a C_α_-RMSD of 1.390 and 1.207 Å, respectively^25^. The main difference between the structures is the linker from amino acids 475–489, located at the surface which adopts a different conformation (Fig. 2c). From a single example of an *Mt*DXPS structure we cannot conclude that this variation is not a result of the crystallization process.

Three inserts in the *Mt*DXPS sequence were found according to a multiple sequence alignment (MSA) of other bacterial DXPS enzymes (Fig. S1). The first insert comprising amino acids 216–220, is in an evolutionary diverse and disordered loop, which is part of the truncated loop in Δ*Mt*DXPS. This region can also not be observed in the wild-type protein crystal structure of other homologues^25–27^. Indeed, the high sequence variability and disorder in the known crystal structures were the reasons to truncate this region for a more stable and more easily crystallizable protein^25–27^.

The other two inserts concern amino acids 498–502 and 523–528 and are located at the protein’s solvent-accessible surface (Fig. 2c). Both insertions occur in all *Mycobacterium* strains, but are in a region of high variability with a pairwise sequence identity of 13.7% and 8.6%, compared to the MSA of 498 other bacterial DXPS, respectively^25^. The first is a loop extension of a ß-sheet turn, while the second is 30 amino acids downstream and forms an α-helix, both are located within domain III, responsible for the extensive contacts with the dimeric interface.

As anticipated, the DXPS active site is highly conserved, and most residues of *Mt*DXPS share the same position across homologues, including key residues shown in mutational studies to be important in catalysis and substrate binding, such as Glu365, Tyr387, Arg415, Asp422, His40 and His71 (Fig. 2b)^25,32,33^. Three seemingly important differences, however, could be identified near the active site of *Mt*DXPS: Lys473, His426 and Ser112. Lys473 plays a significant role in the catalysis and substrate binding, as mutational studies of the corresponding residue on ecDXPS have shown loss of enzymatic activity^25^. Most bacterial DXPS have an arginine in the corresponding position of Lys473. However, the residue is in a conformation similar to the corresponding Arg480/Arg478 of drDXS and ecDXPS structures and the observed *K*_*m*_ for D-GAP is in the same order of magnitude as for these, indicating that the residues could be interchanged without causing major structural changes in the protein^32,34,35^.

His426 is conserved in all bacterial DXPS but adopts a different conformation in the *Mt*DXPS structure. Unlike most bacteria, *M. tuberculosis* has a serine at position 112 instead of a glycine or alanine. The serine at this position creates a steric restriction that prevents His426 from adopting its previously reported conformation, while at the same time enabling the coordination of a water molecule that stabilizes His426 in its new position. The water is highly coordinated, making hydrogen bonds with His426, Ser112, Tyr98 and other water molecules. This network extends to the aminopyrimidine N4’ and the backbone and side chains of Ser425 (Fig. 2d). Despite the overall similarities with other homologues, the structural differences close to the active site provide new information that could be specific for *M. tuberculosis* and will be instrumental for future structure-based drug design endeavors*.*

### ∆*Mt*DXPS with enamine intermediate

In order to further elucidate the structural conformations during catalysis, we also attempted to obtain crystal structures of reaction intermediates. It has previously been shown that the lactyl-conformation of ThDP (LThDP) is stable, while the last steps of the reaction, the decarboxylation of LThDP and the addition of D-GAP, are proceeding fast after binding of D-GAP^20^.

**Figure 3:**
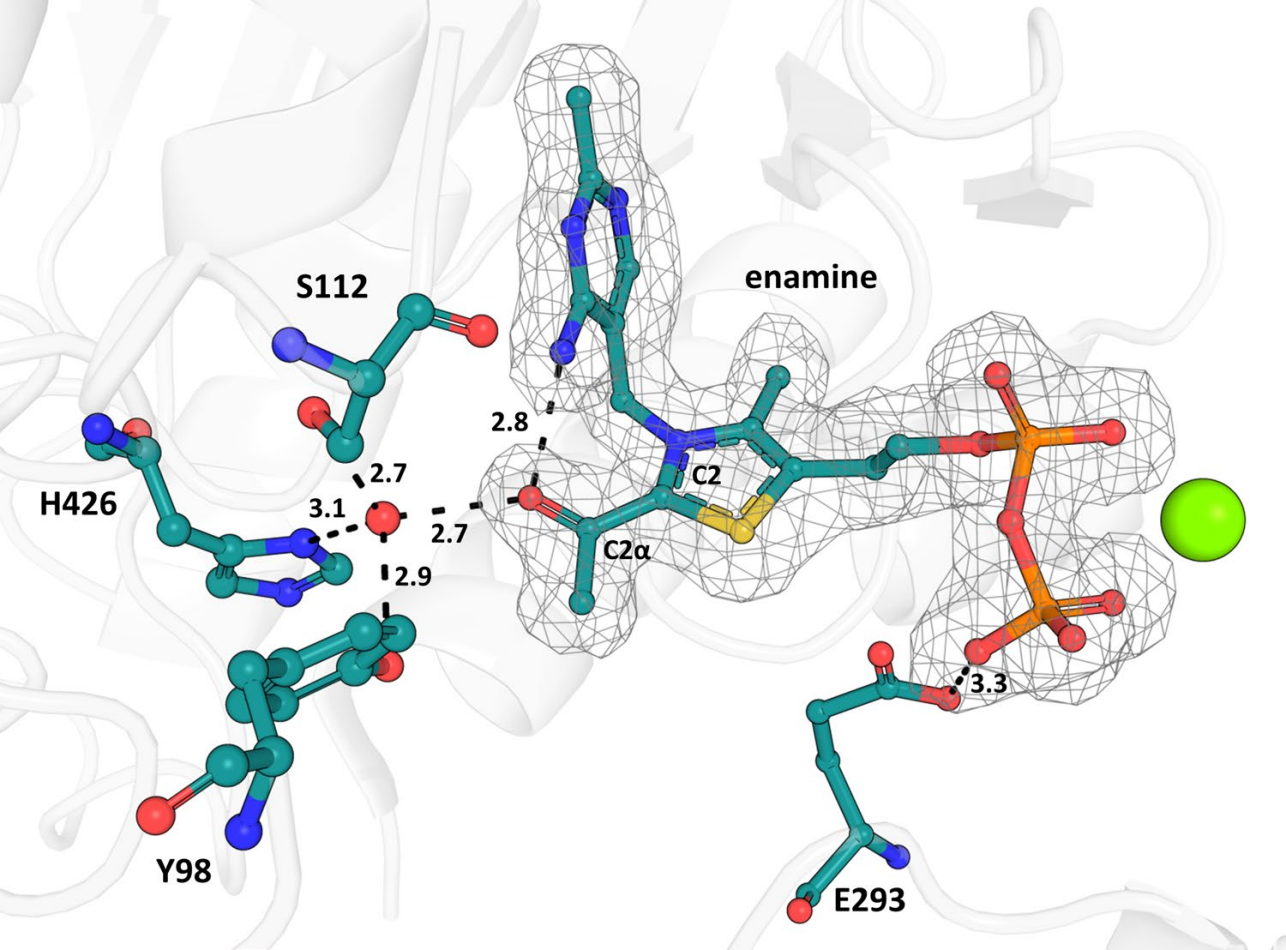
Active site of *∆MtDXPS* with enamine-ThDP intermediate. Polder map density contoured at 3σ in gray wire mesh for the enamine-ThDP intermediate (PDB ID: **7A9G**), calculated using Phenix. Key residues involved in the interaction with the enamine are shown and labeled. Distances are indicated in Ångström.

A structure of Δ*Mt*DXPS simultaneously soaked with pyruvate and D-GAP was solved to a resolution of 1.9 Å (PDB ID: **7A9G**) in space group P2_1_ (Fig. 3). The asymmetric unit contains a homodimer, which adopts a highly similar fold to the Δ*Mt*DXPS holo structure, with an overall RMSD of 0.2 Å. Residues 289–295 are visible in the structure whilst they could not be modeled in the holo structure. In the active site, density could be observed near the C2 of ThDP, consistent with an enamine or acetyl intermediate adduct (Fig. 3)^26^. This decarboxylated form of LThDP shows well-defined electron density with a B-factor of 25 Å^2^ (average in crystal is 22 ± 5 Å^2^). The B-factor is similar to that of the surrounding residues and we can assume that nearly all ThDP ligands in the crystal exist in the enamine or acetyl-form. Since we could not determine whether the enamine has converted to a less reactive acetylThDP state, we will refer to the modeled molecule as the enamine-intermediate. This is the first time the enamine-intermediate was observed in a DXPS structure without growing the crystals in an oxygen-free environment, as was necessary for the first structure of a pyruvate adduct^26^. This is likely the result of the very short time between soaking the crystals with pyruvate and D-GAP and the subsequent flash-cooling, capturing the reaction in an intermediate state.

We found that the enamine–ThDP intermediate is engaged in several unique interactions with Δ*Mt*DXPS not seen in other structures of DXPS reaction intermediates. Glu293 makes hydrogen bonds with the diphosphate moiety of ThDP and the C2α-hydroxyl forms hydrogen bonds with Ser112 and His426 through the highly coordinated water also found in the holo structure (Figs. 2d and 3). In line with other reaction intermediate structures from other DXPS homologues, the N4’ of the aminopyrimidine hydrogen bonds with the C2ɑ-hydroxyl.

**Figure 4:**
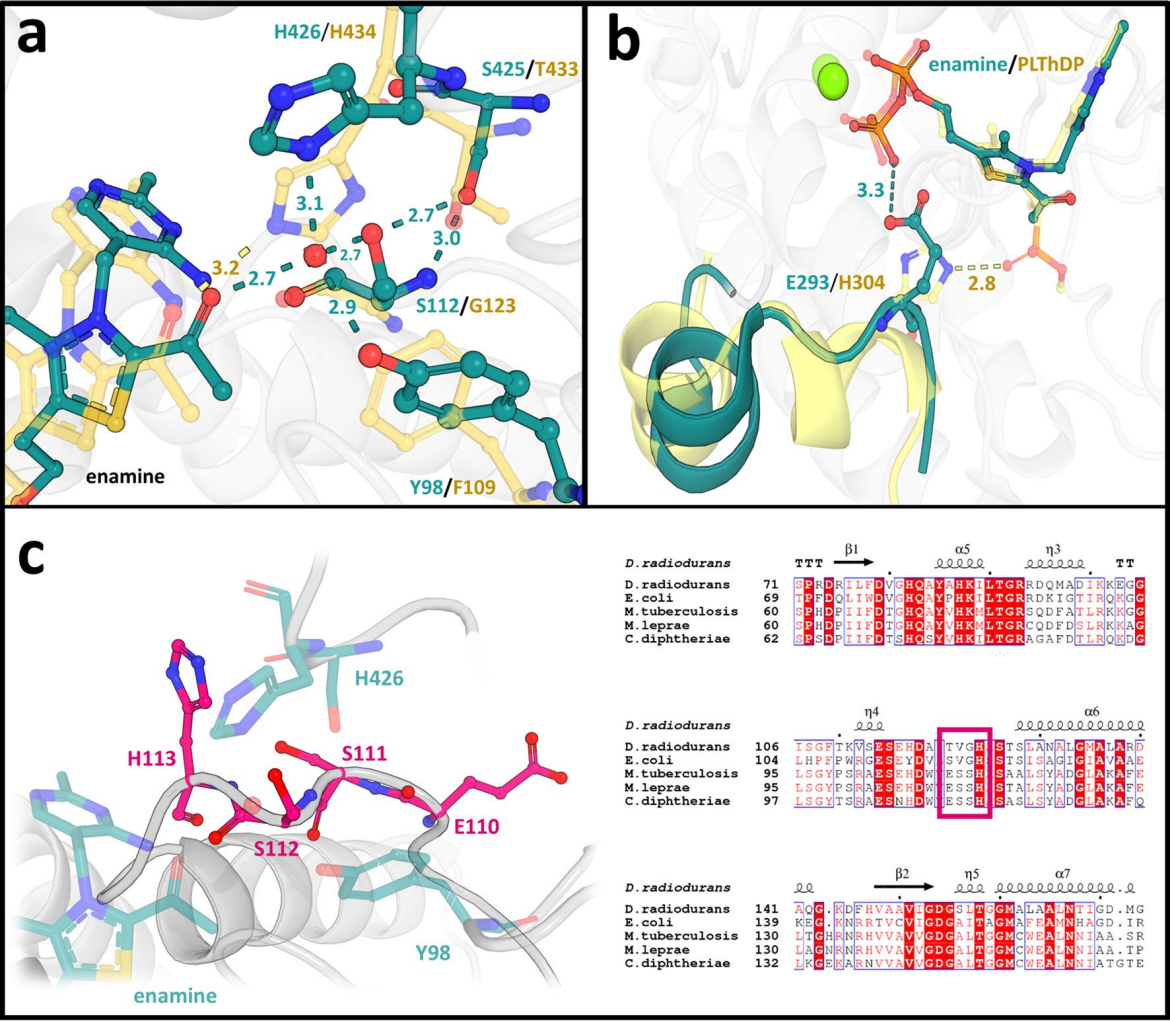
Comparison of drDXPS and MtDXPS enamine intermediate active sites. (**a**) Ser-network/H-bonds stabilizing the enamine intermediate. Residues from MtDXPS are colored in cyan, while the corresponding residues from drDXPS (PDB ID: **6OUW**) are colored in yellow. Hydrogen bonds are shown as dashed lines with distances in Ångström. (**b**) “Fork” motif. The “fork”-like motif found in the intermediate structure is highlighted in cyan, the similar motif in drDXS is colored in yellow. The key difference is the presence of Glu293 in the MtDXPS structure. (**c**) ESSH sequence motif. The ESSH sequence motif found in the ∆MtDXPS structures is shown in pink. The table shows the sequence alignment of bacterial DXPS, with several sequences bearing the ESSH sequence motif shown for representation, highlighted with a pink box.

During the catalytic cycle, ThDP reacts with pyruvate to form stabilized LThDP. Rapid decarboxylation can occur upon D-GAP binding, leading to the formation of the enamine-intermediate. In ∆*Mt*DXPS, the enamine intermediate seems to be stabilized via a highly coordinated water molecule also found in the holo structure. Similarly, the water molecule is coordinated to the enamine intermediate, Ser112, Tyr98 and His426, through a network that further extends to Ser425 and the backbone of Ser112. This network of hydrogen bonds might help delocalize the negative charge of the carbanion during the reaction (Fig. 4a). When the enamine is not present (holo structure, PDB ID: **7A9H**), the position of the enamine-hydroxyl group is occupied by a second water atom that maintains the hydrogen-bond network (Fig. 2d).

In protein structures of drDXPS and ecDXPS, this hydrogen bond (H-bond) network cannot be observed and the enamine-intermediate stabilization is directly maintained through His434 (His426 in *Mt*DXPS) (Fig. 4a). As previously stated, both enzymes have a glycine instead of a serine at position 112 (121 in *E. coli*, 123 *in D. radiodurans*), and consequently no H-bond network can be formed.

The Ser112 is unique for certain bacteria, a multiple sequence alignment with 498 bacterial DXPS sequences showed that only 33 have this amino acid (Table S3). Interestingly, most bacteria that have a serine at this position also have a conserved sequence pattern. The pattern starts with Glu110, followed by Ser111, Ser112 and His113, which we called ESSH sequence motif (Fig. 4c). His 113 is essential, with 100% conservation, but the rest of the sequence is in a variable region with only 43.8% similarity. All Mycobacteria*,* together with the closely related Corynebacteria and a few other species, bear this sequence motif, highlighting that this different mechanism could be specific for those bacteria (Table S3).

### Residues near the active site set MtDXPS apart from other DXPS and could account for its lower activity

Two crystal structures from *D. radiodurans* were recently published by Drennan and coworkers, in which a “fork” and a “spoon” motif were introduced. These motifs adopt different conformations during the catalytic cycle^26,29^. The “fork” and “spoon” motifs are two loops adjacent to each other, in positions 292–306 and 307–319 (drDXPS). The corresponding amino acids in *Mt*DXPS are 283–299 and 300–312, for the “fork” and “spoon”-motif, respectively. The “fork” motif in the *Mt*DXPS structure seems to adopt a slightly different fold and does not include His304 (drDXS) (Fig. 4b). In both structures of *Mt*DXPS, Histidine 296, which corresponds to His304 in drDXPS, could not be observed. This His304 is part of the active site and has previously been identified by mutational studies as important for the catalysis through a stabilization of both LThDP and the closed conformation of the enzyme^19,26^. Experiments have demonstrated a 90% reduction of DXPS activity when His304 is mutated to alanine^32^. In both structures of *Mt*DXPS, no histidine making similar interactions was observed. In chain A of our intermediate structure (PDB ID: **7A9G**, chain A), part of the residues that would correspond to the fork motif orient in a similar, but slightly different fold, resulting in the position of catalytically important His304 in drDXPS being occupied by Glu293, which forms hydrogen bonds with the diphosphate group of ThDP (Fig. 4b). These residues are disordered in chain B and the holo structure. Since His304 (drDXPS) stabilizes the LThDP by interacting with its carboxylate group, the presence of Glu293 in that location in *Mt*DXPS and its interaction with the diphosphate may lead to a lower stabilization of the LThDP intermediate and the closed conformation of the protein, potentially contributing to the lower catalytic activity.

### GAP binding site

**Figure 5:**
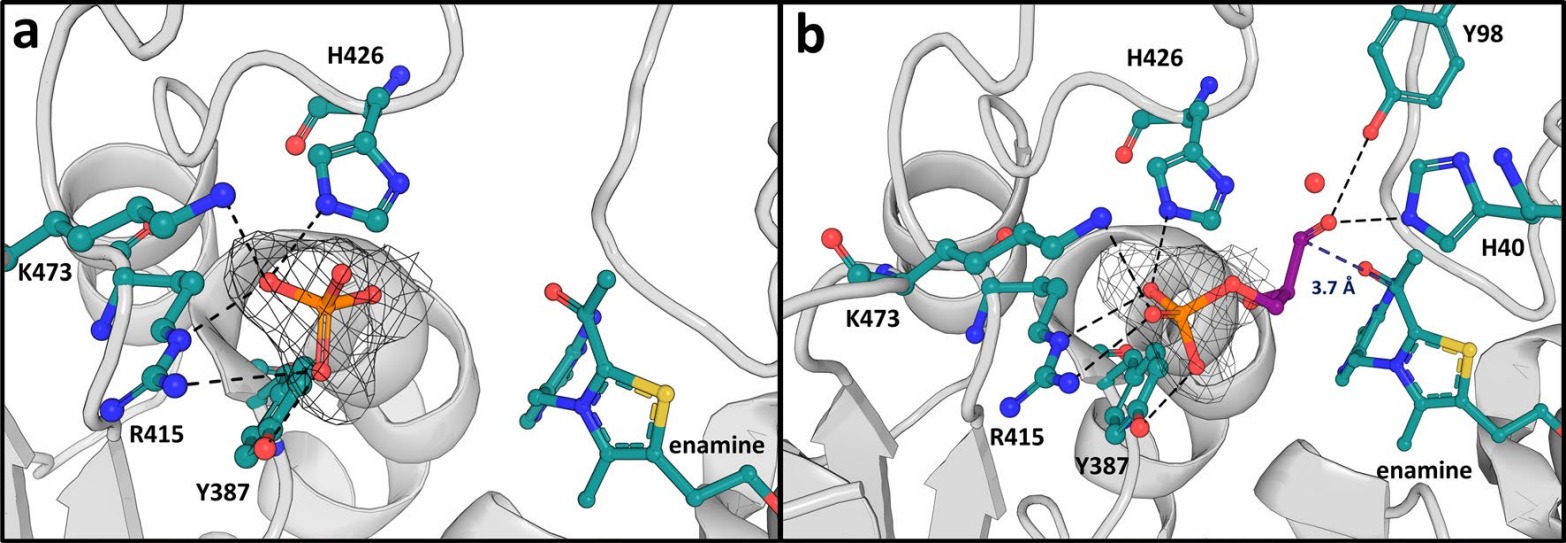
∆*Mt*DXPS D-GAP binding site with modeled phosphate and docked D-GAP. (**a**) ∆*Mt*DXPS structure (PDB ID: **7A9G**) with polder map density contoured at 3σ in gray wire mesh for the phosphate. Key residues involved in the interaction with the phosphate are shown in stick representation and labeled. Hydrogen bonds are shown as black, dashed lines. (**b**) D-GAP molecule docked into the D-GAP binding site of ∆*Mt*DXPS after removal of the phosphate. The polder density map of the phosphate present in the crystal structure is shown to highlight the similar orientation and interactions of the 3-phosphate moiety of D-GAP to the modeled phosphate. The residues predicted to interact with the docked D-GAP are shown in stick representation and are labeled. Predicted hydrogen bonds are shown as black, dashed lines, while the distance between the D-GAP C3 and the enamine intermediate 2C_α_ are shown as blue, dashed lines.

Observation of the second substrate D-GAP was more difficult in the protein crystals soaked with both substrates. This was expected, as we did not trap the enzyme in a catalytic state for instance, by a dead-end substrate, but instead exposed the protein crystals to a solution allowing catalytic activity. This resulted in different catalytic states in the same protein crystal. However, it was possible to identify the electron density of a phosphate-like moiety and model it with an elevated B-factor of 49.3 Å^2^ (Fig. 5a). While the C3-body of D-GAP cannot be built with confidence, the crystallization conditions do not contain free phosphate, indicating that the observed phosphate density could be provided by a partially disordered D-GAP molecule. The electron density for this moiety can be observed clearly in chain B of the homodimer, while it is more diffuse in chain A. The phosphate is located in close proximity to the enamine intermediate and forms hydrogen bonds with Tyr387, Arg415, His426 and Lys473 with distances between 2.8 and 3.4 Å. The interacting residues correspond to the residues identified to make up the D-GAP binding site in previous studies, determined by molecular docking or kinetic studies and alanine-scanning^20,35^.

Irrespective of the presence of a fully ordered D-GAP, the density of the putative phosphate group provides a reasonable starting point to model and dock the complete D-GAP substrate (Fig. 5b). The substrate molecule docks well into the binding site, with its 3-phosphate moiety superimposing with the observed density and making the same interactions with Tyr387, Arg415, His426 and Lys473 as the phosphate visible in the electron density. The carbonyl group interacts with Tyr98 and His40, which helps to orient the C3-body in an ideal position for the nucleophilic attack of the C2-carbanion of the enamine intermediate (Fig. 5b).

### Docking of *Mt*DXPS inhibitors from the literature

#### Hydroxybenzaldoximes

With the first protein crystal structure of a pathogenic DXPS homologue it is now possible to understand the binding of inhibitors of DXPS by in-silico docking. As an example, the class of trihydroxybenzaldoximes was chosen. This compound class is one of very few reported D-GAP-competitive inhibitors and does not compete with pyruvate^36^. With the first crystal structure that resembles the conformation of the active site during the D-GAP addition, and the additional hint for the D-GAP position, we were interested in the interactions of this compound class with the protein.

**Figure 6:**
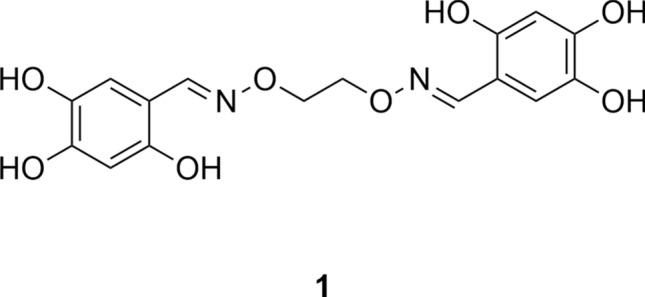
Example structure of the literature known DXS inhibitor class of hydroxybenzaldoximes **1**^36^.

The class of trihydroxybenzaldoximes was published first as inhibitors of DXPS by Bartee et al.^36^. The inhibitors were developed based on the similarity with the substrates of DXPS. One side was kept as analogue of pyruvate, the first and specific substrate of DXPS, the other side of the chain was varied with aryl residues, based on previous findings that DXPS has a high substrate promiscuity and is able to accept several different acceptor substrates^37,38^. During inhibitor development, Bartee et al. discovered the high activity of compound **1**, which had no pyruvate substitute, but was rather a symmetrical oxime (Fig. 6).

**Figure 7:**
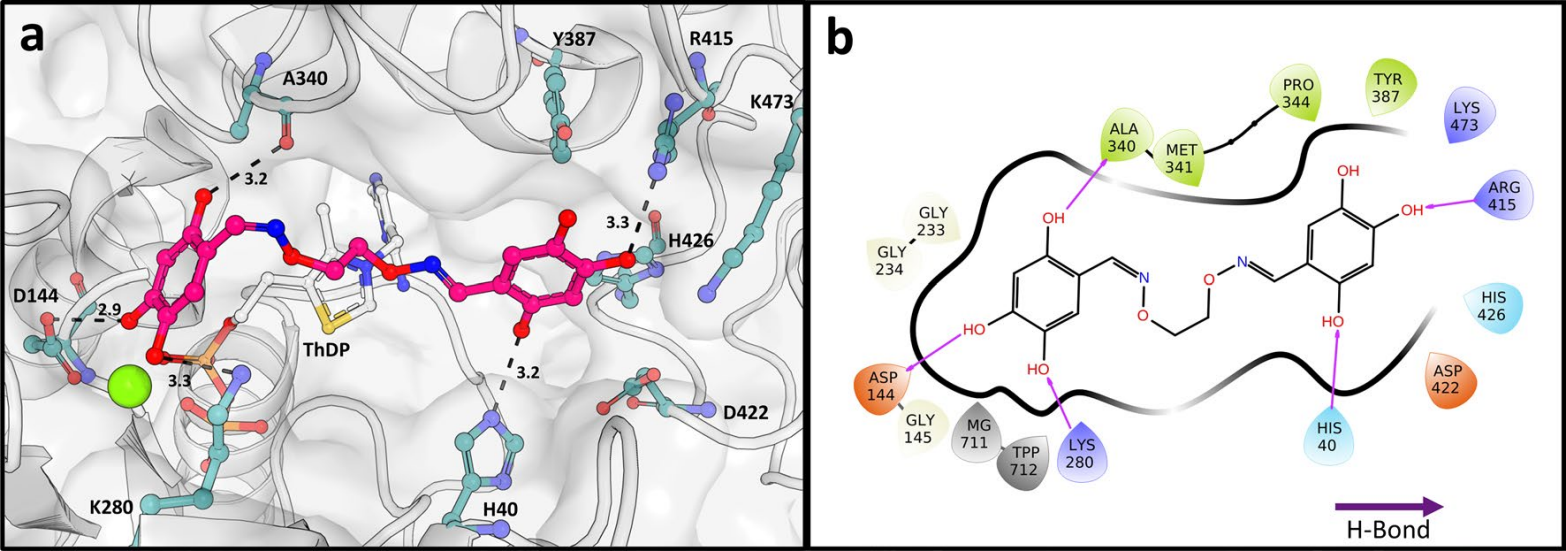
Docking of the hydroxybenzaldoxime 1 to ∆*Mt*DXPS. (**a**) Hydroxybenzaldoxime 1 docked in the active site of ∆*Mt*DXPS with cofactor ThDP bound (PDB ID: 7A9H). The pose of the docked molecule with the highest affinity score, as calculated by HYDE, is shown in pink and the predicted interacting residues are shown in stick presentation and are labeled. Hydrogen bonds are shown as black, dotted lines with distances indicated in Ångström. (**b**) 2-Dimensional ligand interaction plot of Hydroxybenzaldoxime 1 with ∆*Mt*DXPS.

We docked the reported class of hydroxybenzaldoximes to our protein structure, using the docking software SeeSAR. The compound with the highest biological activity (*K*_i_ 1 ± 0.2 µM) against DXPS from *D. radiodurans*, compound **1**, docks well into the active site (Fig. 7). The docking resulted in 20 poses ranked using affinity scores, as calculated by HYDE. The docking pose with the highest affinity score was chosen and shows interactions of the hydroxyl groups from one part of the molecule with the amino acids of the D-GAP binding site, while the linker between the two similar warheads is spanning over the active site. Besides hydrogen bonds with the protein, the second part can form chelating interactions with the bound Mg^2+^-ion. This metal interaction is remarkable, as it allows the compound to interact as the sixth coordination partner of the cation, while not competing with the diphosphate group of the ThDP cofactor, but instead additionally forming an H bond with the terminal β-phosphate. When docked to the protein structure with the bound intermediate, the inhibitor adopts a similar pose, shielding the enamine-ThDP from the solvent. This enclosure of the cofactor or intermediate is a possible explanation of the observed distinctive inhibition mode, competitive to D-GAP and noncompetitive to pyruvate^36^.

## Discussion

DXPS is one of the most important enzymes of the 2-*C*-methyl-d-erythritol 4-phosphate (MEP)-pathway and is a key target for the development of new anti-infective drugs to treat infections with multidrug-resistant bacteria. Here, we have applied our previously reported truncation approach and removed a disordered internal loop, to successfully crystallize the DXPS from an important anti-infective target, namely *M. tuberculosis*.

The Δ*Mt*DXPS structures show a high degree of similarity to known structures. However, our work proposes a different stabilization mechanism of the enamine intermediate in *Mt*DXPS when compared to the enamine-intermediate structure of drDXPS. We propose that the presence of the highly coordinated water, found in both the holo and enamine-intermediate structures, interacting with Ser112, His426 and Y98, and the different conformation of H426 might help delocalize the carbanion charge, yielding a more stable intermediate. Even though mutational studies with *E. coli* and *D. radiodurans* have shown that the corresponding residues of H426 (H434 in drDXPS and H431 in ecDXPS) are not essential for catalysis and may therefore mainly contribute to substrate binding affinity, the different conformation of this residue in the Δ*Mt*DXPS structures suggests that it plays a different role for *M. tuberculosis* DXPS^32^.

Compared with other DXPS enzymes, which are reported to have *k*_cat_ values in the range of 0.5–25 s^–1^, the *Mt*DXPS possesses the slowest reaction kinetics for a DXPS homologue described to date, with a *k*_*cat*_ value in the range of 0.005 s^–1^ ^8,39^. The distinct enamine-intermediate stabilization mechanism found in Δ*Mt*DXPS might explain its slower reaction rate. The Ser112 present in all *Mycobacterium* could be an important factor that explains this difference in the stabilization mechanism, however, there are no catalytic data from any other homologue containing a serine in this position reported to date. Obtaining kinetic information of such homologues could support these observations. In addition, to confirm whether the serine has any impact on the observed difference in kcat, the position should be mutated in the *Mt*DXPS. The same holds true for the other amino acids involved in the hydrogen-bond network of the water molecule (Tyr 98, His426 and the ESSH sequence motif). Furthermore, these positions could be inserted into other, well characterized DXPS enzymes, such as drDXPS or ecDXPS, in order to obtain valuable information about their potential effect on the reaction rate.

Independent of the direct effect of the serine or the sequence motif on the catalytic activity, species with these features are more likely to have a slightly larger active site, due to the different rotameric state of His426 (Fig. 4a). By targeting this pocket or introducing a group that can interact with the H-bonding network in a similar manner to the intermediate, it might be possible to develop DXPS inhibitors selectively targeting *M. tuberculosis* over other species.

Distinct from previous studies performed on drDXPS, which found a fork-spoon motif that is positioned to open and close the active site based on substrate binding^19,26^, a similar but divergent structural feature was found for *Mt*DXPS. This ‘fork-like’ motif utilizes a different residue for the interaction with the cofactor, potentially leading to a lesser degree of fixation of the active site conformation.

As of yet, we could not obtain a structure of *Mt*DXPS with a fully ordered spoon-fork motif. This leaves the question open whether His296 interacts, in any conformation, with the active site of *Mt*DXPS. If His296 is permanently located outside of the active site and the corresponding position is taken by Glu293, which makes different interactions with the diphosphate moiety of ThDP, this could also explain the lower enzymatic activity of *Mt*DXPS. As mentioned, the mutation of His304 to alanine in drDXPS leads to a 90% reduction in activity^32^. Similar mutational studies in *Mt*DXPS could be performed to evaluate their impact on the enzymatic activity, such as mutating Glu293 and His296 to alanine, as these might be crucial for the observed differences in the spoon-fork motif. Since drDXPS and ecDXPS do not have a corresponding amino acid in this position (Fig. S1), inserting it in their sequences could grant further insight into its role. A *Mt*DXPS structure containing the LThDP mimic PLThDP could provide valuable insights into the conformation and interactions of the fork-like motif pre-decarboxylation and its potential effect on the catalytic activity of *Mt*DXPS. Unfortunately, thus far, we were unable to obtain such a structure. However, the availability of high-resolution crystals of *Mt*DXPS allows a more complete exploration of the molecular mechanisms behind the lower DXPS activity of *Mt*DXPS, which will be the subject of future research.

Furthermore, this was the first time that phosphate-like electron density could be observed near the predicted D-GAP binding site in a DXPS structure, providing new evidence of the putative binding site and residues involved in substrate stabilization. While the observed density and docking of the D-GAP substrate do not provide definitive evidence of D-GAP binding, these results fit well with expectations of D-GAP substrate binding^20,35^. Chain A of the intermediate structure shows a partially folded, fork-like motif with Glu293 interacting with ThDP and only weak density in the D-GAP binding site. The fork-like motif in chain B is disordered but phosphate-like density can be observed in the D-GAP binding site. It could be postulated that the enzyme can adopt both the open and closed conformation post-decarboxylation depending on the presence of D-GAP/phosphate. Bound D-GAP/phosphate would induce an open conformation whilst the fork-like motif closes in the absence of D-GAP.

As mentioned, our group attempted a ligand-based approach for the discovery of inhibitors of DXPS using a drDXPS crystal structure and a homology model of *Mt*DXPS derived from that structure. This yielded compounds that showed activity against drDXPS but less so against *Mt*DXPS. The homology model active site differs from the mtDXPS crystal structure active site in the same manner as the drDXPS active site differs from the MtDXPS, which can be explained by the fact that it was modeled using the drDXPS crystal structure. The conformation of the spoon-fork motif, the orientation of His426, the absence of the coordinated water and the structural position of Glu293 of the homology model match the drDXPS crystal structure, whilst the *Mt*DXPS crystal structure shows that these are distinct (data not shown). Since these amino acids were proposed to form several key interactions with the compounds designed in our previous paper^22^, it could explain why they did not have the expected activity against *Mt*DXPS. This highlights the importance of obtaining crystal structures as computationally generated models do not always reflect the real conformations of proteins, leading to inhibitor design using incorrect data.

In summary, the successful use of the loop truncation for the crystallization of DXPS from a different species, provides a solid platform that can extend the structural study of DXPS homologues. All structural differences obtained, such as the ones found for *Mt*DXPS, can then assist in the development of new antibiotics with a high specificity for species that are already drug-resistant, such as *M. tuberculosis*. Additionally, our results provide a new opportunity to investigate and improve previously identified inhibitors of *Mt*DXPS by docking them into the experimentally determined structures, allowing for the elucidation of SARs and facilitating the design and hit-to-lead optimization of TB-specific inhibitors. The next step will be to obtain co-crystal structures with inhibitors bound to the enzyme.

## Methods

### Expression and purification of *Mt*DXPS

The design of a pET22b plasmid hosting the native *Mt*DXPS gene with an N-terminal His-Tag is reported elsewhere^40^. The plasmid was transformed into *E. coli* BL21 (DE3) cells, which were grown at 37 °C. At an OD_600_ of ~ 0.6, protein expression was induced by the addition of isopropyl β-d-thiogalactopyranoside (IPTG) to a final concentration of 0.1 mM, and the cells were incubated at 18 °C overnight. The bacteria were harvested by centrifugation and resuspended in lysis buffer [50 mM Tris–HCl pH 8.0, 300 mM NaCl, 10 mM imidazole, 5 mM β-mercaptoethanol (β-ME), 5 mM MgCl_2_, 2.5 U/mL benzonase, 1 tablet/200 mL *Complete* Protease inhibitor, EDTA free (Roche)]. The cells were lysed using a Microfluidizer M-110P, and the lysate clarified by centrifugation for 45 min at 18,000*g*. The lysate was filtered using a 0.45 µm syringe filter, loaded onto a metal-affinity HisTrap HP 5 mL column (GE Healthcare) and eluted using a linear gradient of imidazole from 10 to 500 mM. Protein-containing fractions were pooled and concentrated using VivaSpin ultrafiltration devices with a MWCO of 30 kDa. The concentrated sample was further purified by gel filtration on a HiLoad 16/600 Superdex 200 pg column equilibrated in 20 mM Bis–Tris-Propane, pH 7.5; 300 mM NaCl, 1 mM MgCl_2_, 0,5 mM TCEP-HCl.

### Expression and purification of ∆*Mt*DXPS

The truncated *Mt*DXPS gene was obtained commercially as a synthetic gene and cloned into a pETM-11 plasmid using the NcoI and HindIII restriction sites. Expression, lysis and the initial IMAC purification were performed as above for full-length *Mt*DXPS. The protein-containing fractions were subsequently combined and diluted with a low-salt buffer consisting of 50 mM HEPES pH 8.0, 5% glycerol, 5 mM dithiothreitol (DTT) and 100 µM MgCl_2_ to a conductivity of 8 mS/cm. The solution was then loaded on a Resource Q anion exchange column and eluted with a linear NaCl gradient from 0 to 1 M. The protein-containing fractions were pooled and purified by gel filtration on a HiLoad 16/600 Superdex 200 pg column equilibrated with 20 mM HEPES pH 8.0, 250 mM NaCl, 5% glycerol, 5 mM DTT. The protein-containing fractions were concentrated to 5.5 mg/mL using a Vivaspin centrifugal concentrator (MWCO 30 kDa, Sartorius), and the His-tag was cleaved by TEV-protease digestion at 10 °C overnight. Removal of the tag and protease was achieved by reversed IMAC chromatography, and the protein was purified again by gel filtration on a HiLoad 16/600 Superdex 200 pg column using 20 mM MOPS pH 7.50, 200 mM NaCl, 5% Glycerol, 2 mM DTT as buffer. The purified protein was concentrated to 10 mg/mL in a Vivaspin centrifugal concentrator (MWCO 10 kDa, Sartorius).

### Protein crystallization and structure determination

A high-throughput crystallization robot (Mosquito, SPT Labtech) was used to perform sitting-drop vapor-diffusion screenings for crystallization conditions for Δ*Mt*DXPS. Prior to screening, 1 mM of ThDP and 2 mM MgCl_2_ were added to the protein solution (10 mg/mL) and then incubated at 4 °C for 3 h. The mixture was then screened against commercially available sparse-matrix screening kits (PACT premier, JSCG plus and MORPHEUS; Molecular Dimensions) at 18 °C. 200 nL of protein solution and 200 nL of crystallization buffer were equilibrated against 50 μL of reservoir solution. Initial crystals were obtained after 2 days in MORPHEUS conditions B2 (0.1 M MES/imidazole pH 6.5, 10% w/v PEG 8000, 20% v/v ethylene glycol, 0.3 M sodium fluoride, 0.3 M sodium bromide and 0.3 M sodium iodide) and C2 (0.1 M MES/imidazole pH 6.5, 10% w/v PEG 8000, 20% v/v ethylene glycol, 0.3 M sodium nitrate, 0.3 M disodium hydrogen phosphate and 0.3 M ammonium sulfate) as well as PACT premier D2 (0.1 M MMT-buffer pH 5.0, 25% PEG1500). The structures described in this manuscript were obtained from crystals grown in condition PACT premier D2. The crystals were cryo-protected using reservoir solution supplemented with 30% (v/v) PEG 400 and flash-cooled in liquid nitrogen.

To obtain Δ*Mt*DXPS with reaction intermediate, crystals grown in the same condition were harvested, soaked for one minute in the above-mentioned cryo-protecting solution supplemented with 1 mM of D-GAP and 1 mM of pyruvate and subsequently flash-cooled in liquid nitrogen. X-ray diffraction data for the Δ*Mt*DXPS structures were collected on beamline P13 operated by EMBL Hamburg at the PETRA III storage ring (DESY, Hamburg, Germany) at 100 K. Data indexing, integration and scaling was performed using XDSAPP^41^ and AIMLESS^42^ from the CCP4^43^ software package. The structure was solved using the MOLREP^44^ software in CCP4 using the previously solved drDXPS homologue (PDB ID: **2O1X**) as the reference model. The resulting models were then subjected to iterative cycles of model building and refinement with COOT^45^ and REFMAC5^46^.

The structures were deposited in the PDB with accession codes **7A9H** and **7A9G**, corresponding to the holo and reaction intermediate structures, respectively. Table S2 contains the data collection and refinement statistics.

### Enzymatic assay

The DXPS activity was analyzed at RT as previously reported, with minor modifications^40,47^. A continuous kinetic photometric assay was used to measure DXPS activity. NADPH depletion by the downstream IspC enzyme was determined in a microplate reader (PHERAstar, BMG Labtech) by monitoring the decrease in absorbance at 340 nm. Total assay volume was 60 µL, containing 200 mM HEPES pH 8.0, 2 mM DTT, 1 mM MgCl_2_, 0.3 mM NADPH and 1.5 µM IspC (from *E.coli*, expressed and purified in-house according to a literature procedure)^48^. The amount of DXPS used in the assays was determined experimentally by a dilution series of the enzyme. These were 5 µM and 2 µM for ∆*Mt*DXPS and *Mt*DXPS, respectively, as they showed the highest linear reaction velocity without observable substrate depletion over a time range of 30 min. The reaction was monitored at RT for 30 min after addition of the substrate(s) and 1 min of centrifugation (2000 rpm). To determine the corresponding *K*_m_ values, the compounds ThDP, pyruvate and D-GAP were used in varying concentrations. If a substrate or cofactor was kept constant, a concentration of 0.2 mM was used for ThDP, 0.5 mM for pyruvate and 2 mM for D-GAP.

Blank correction and linear fitting of the absorption data was performed using the program Origin 2019 (OriginLab). The initial velocities obtained were plotted against the substrate concentrations, and the *K*_m_ values were determined by nonlinear curve fitting using the Michaelis–Menten model of the enzyme kinetics add-on of Origin2019.

### Thermal shift assay (TSA)

Thermal shift analyses were performed using an ABI StepOneplus RT-PCR instrument. The samples were measured in white 96-well plates. Denaturation was achieved using a continuous heating rate of 1 °C/min from 20 to 95 °C. The total sample volume was 25 µL, consisting of 20 µL TSA buffer (20 mM Tris–HCl, pH 8.0; 100 mM NaCl, 5 mM MgCl_2_), 2.5 µL protein solution and 2.5 µL dye (Sypro Orange, Sigma-Aldrich). The optimal concentrations were experimentally determined. A final concentration in the plate of 1.5 µM protein and 5× SYPRO Orange yielded the best signal-to-noise ratio. All measurements were performed in duplicate.

### LC–MS

All ESI–MS-measurements were performed on a Dionex Ultimate 3000 RSLC system using an Aeris Widepore XB-C8, 150 × 2.1 mm, 3.6 µm dp column (Phenomenex, USA). Separation of 1 µL sample was achieved by a linear gradient from (A) H_2_O + 0.1% formic acid (FA) to (B) ACN + 0.1% FA at a flow rate of 300 µL/min and 45 °C. The gradient was initiated by a 0.5 min isocratic step at 2% B, followed by an increase to 75% B in 10 min to end with a 3 min step at 75% B before re-equilibration with initial conditions. UV spectra were recorded by a DAD in the range from 200 to 600 nm. The LC flow was split to 75 µL/min before entering the maXis 4G hr-ToF mass spectrometer (Bruker Daltonics, Bremen, Germany), using the standard Bruker ESI source. In the source region, the temperature was set to 200 °C, the capillary voltage was 4000 V, the dry-gas flow was 5.0 L/min and the nebulizer was set to 1.0 bar. Mass spectra were acquired in positive ionization mode ranging from 600 to 1800 m/z at 2.5 Hz scan rate. Protein masses were deconvoluted by using the Maximum Entropy algorithm (Copyright 1991–2004 Spectrum Square Associates, Inc.).

### Modeling and docking

The computer program SeeSAR, version 10.3.1 from BioSolveIT was used to generate the docking poses and calculate binding affinities. The software uses the FlexX docking algorithm for the placement of ligands^49^. The affinities are estimated using the HYDE scoring function, which calculates the binding affinities based on the hydration differences between the bound and unbound state of the molecule^50,51^. The binding site was chosen around the ThDP ligand, extending to the residues Tyr387, Arg415 and Lys473, which bind the D-GAP substrate. Sequence numbering is based on the Uniprot sequence file with the code **P9WNS3**. Analysis and visualization of the results were done using the program StarDrop, version 6.6.7.25378 from Optibrium.

## Supplementary Information

Supplementary Information.

## Data Availability

***Accession numbers*** Protein structures are deposited in the PDB archive with the PDB ID: **7A9G** and **7A9H**. All raw data presented in this publication are available upon request from Matthew R. Groves, email: m.r.groves@rug.nl.

## Abbreviations

DMADP: Dimethylallyl diphosphate
DOXP: 1-Deoxy-d-xylulose 5-phosphate
DXPS: 1-Deoxy-d-xylulose 5-phosphate synthase
IDP: Isopentenyl diphosphate
IMAC: Immobilized metal ion affinity chromatography
LThDP: C2α-lactylThDP
MEP-pathway: 2-*C*-Methyl-d-erythritol 4-phosphate pathway
MSA: Multiple sequence alignment
SAR: Structure–activity relationship
TB: Tuberculosis
TDR-TB: Totally drug-resistant TB
ThDP: Thiamine diphosphate
WHO: World Health Organization
