# Supplementary material for: First crystal structures of 1-deoxy-d-xylulose 5-phosphate synthase (DXPS) from Mycobacterium tuberculosis indicate a distinct mechanism of intermediate stabilization
Source: Sci Rep. 2022 May 4;12:7221. doi: 10.1038/s41598-022-11205-9 (PMC9068908; doi:10.1038/s41598-022-11205-9)
Supplement: Supplementary file 1 — Supplementary Information. [file 41598_2022_11205_MOESM1_ESM.docx]

# Supplemental Information: **First crystal structures of 1-deoxy-D-xylulose 5-phosphate synthase (DXPS) from *Mycobacterium tuberculosis* indicate a distinct mechanism of intermediate stabilization.**

Robin M. Gierse ^a,b,c^, Rick Oerlemans ^d^, Victor O. Gawriljuk ^e^, Eswar Reddem ^c,d^, Alaa Alhayek ^a,b^, Dominik Baitinger ^a^, Harald Jakobi ^f^, Bernd Laber ^f^, Gudrun Lange ^f^, Anna K.H. Hirsch* ^a,b,c^ and Matthew R. Groves* ^d^

[Sequence alignment of DXPS from *D. radiodurans*, *E. coli* and *M. tuberculosis*](#_heading=h.30j0zll) [2](#_heading=h.30j0zll)

[LC-MS analysis of ∆mtDXPS](#_heading=h.1fob9te) [3](#_heading=h.1fob9te)

[Enzyme stability](#_heading=h.3znysh7) [4](#_heading=h.3znysh7)

[Enzyme kinetics](#_heading=h.tyjcwt) [5](#_heading=h.tyjcwt)

[MEP pathway enzymes](#_heading=h.1t3h5sf) [6](#_heading=h.1t3h5sf)

[Collection and refinement statistics](#_heading=h.17dp8vu) [7](#_heading=h.17dp8vu)

[Sequences](#_heading=h.3rdcrjn) 9

### **Sequence alignment of DXPS from *D. radiodurans, E. coli* and *M. tuberculosis***


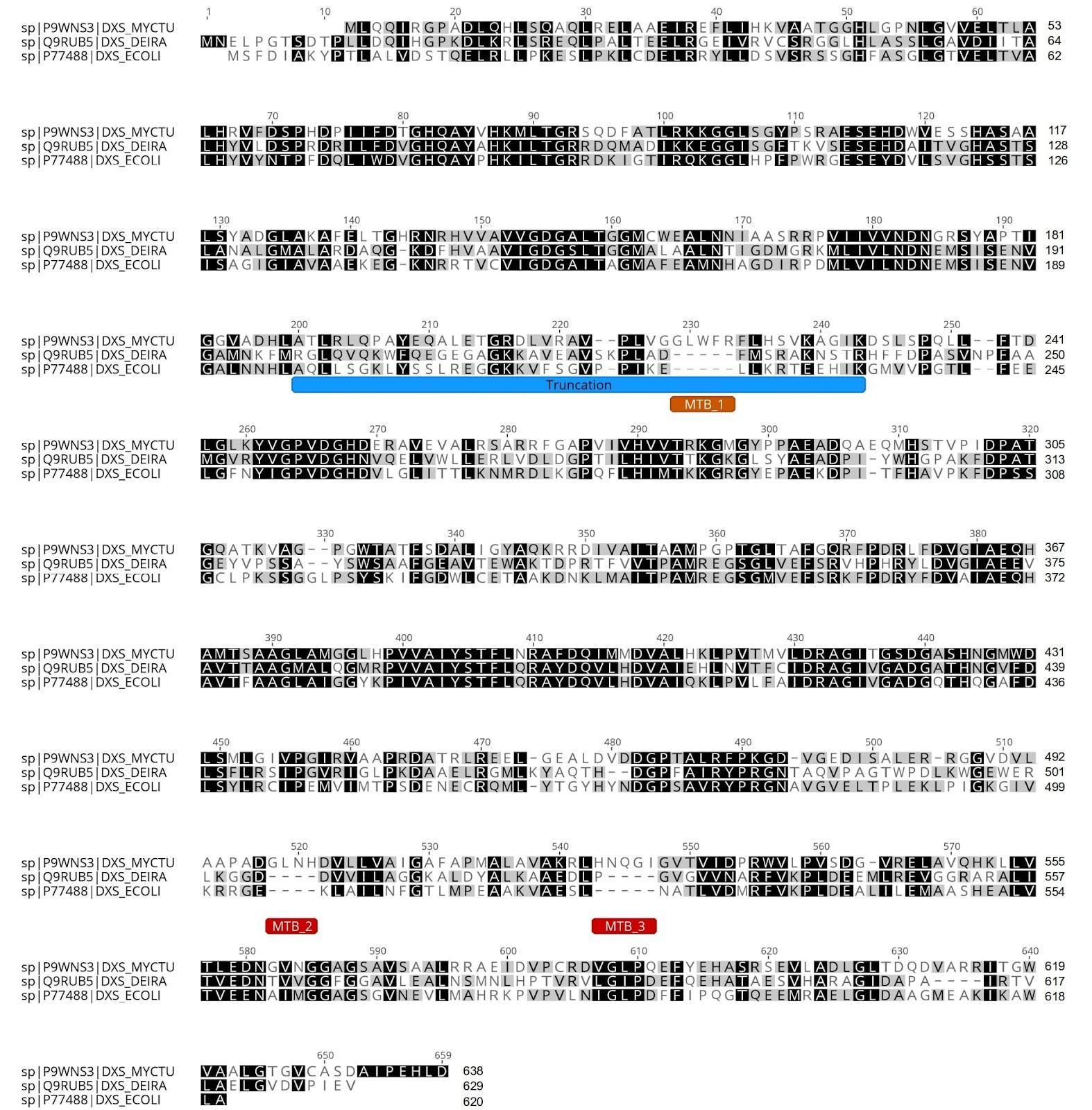


**Figure S1:** Sequence alignment of the three crystallized DXPS homologues from *D. radiodurans, E. coli* and *M. tuberculosis*. Sequence inserts of *M. tuberculosis* are annotated in red, the site of truncation in blue.

### **LC-MS analysis of ∆mtDXPS**


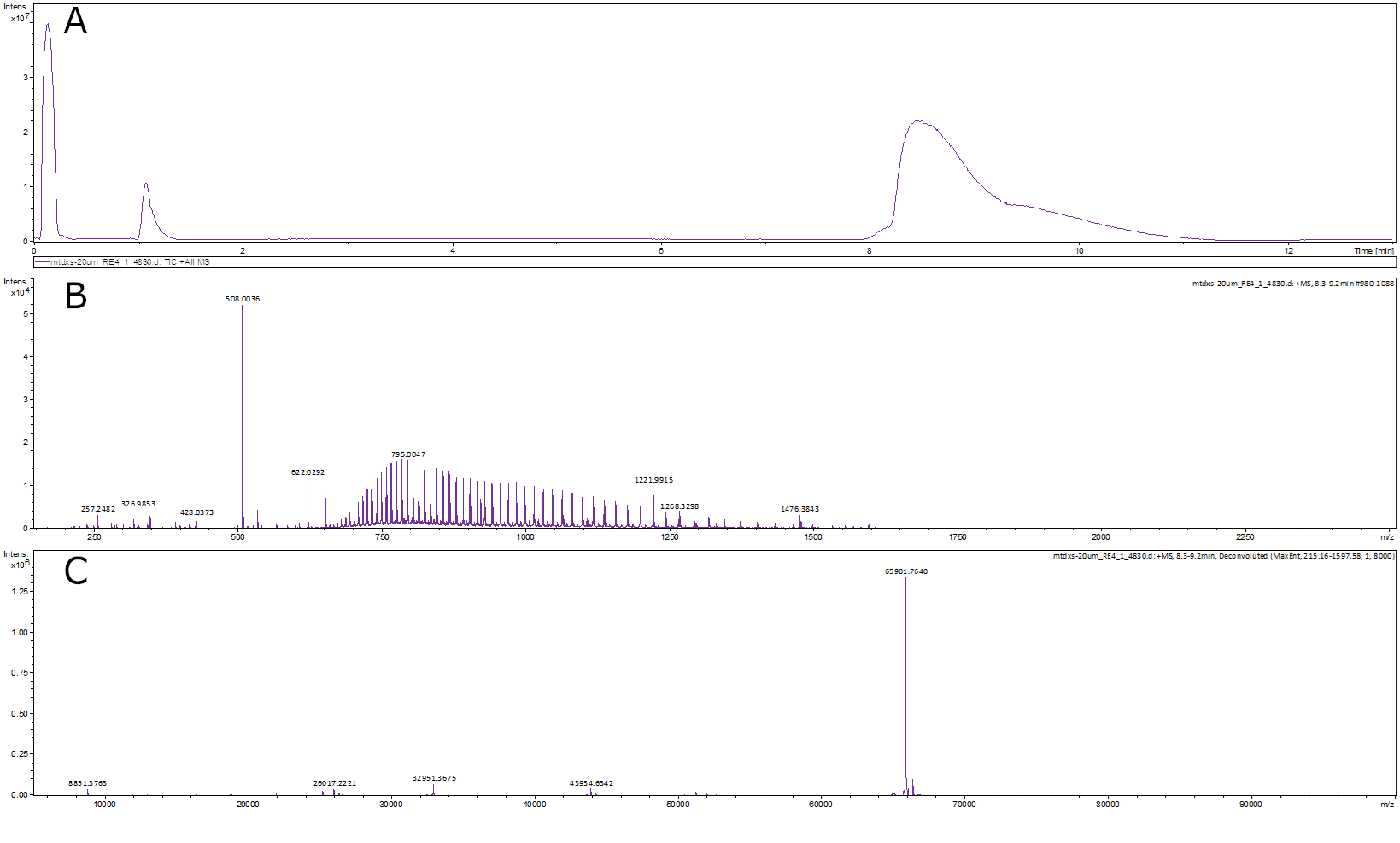


**Figure S2:** LC-MS analysis of a 20 µM sample of ∆mtDXPS. **A:** Total ion count (TIC) spectra of the sample. **B:** LC-MS spectrum of the peak at minute 8.3 to 9.2. **C:** Deconvoluted mass spectrum of the peak from minute 8.3 to 9.2. The full mass of the mtDXPS protein can be observed with a m/z of 75901.67.

### **Enzyme stability**

###


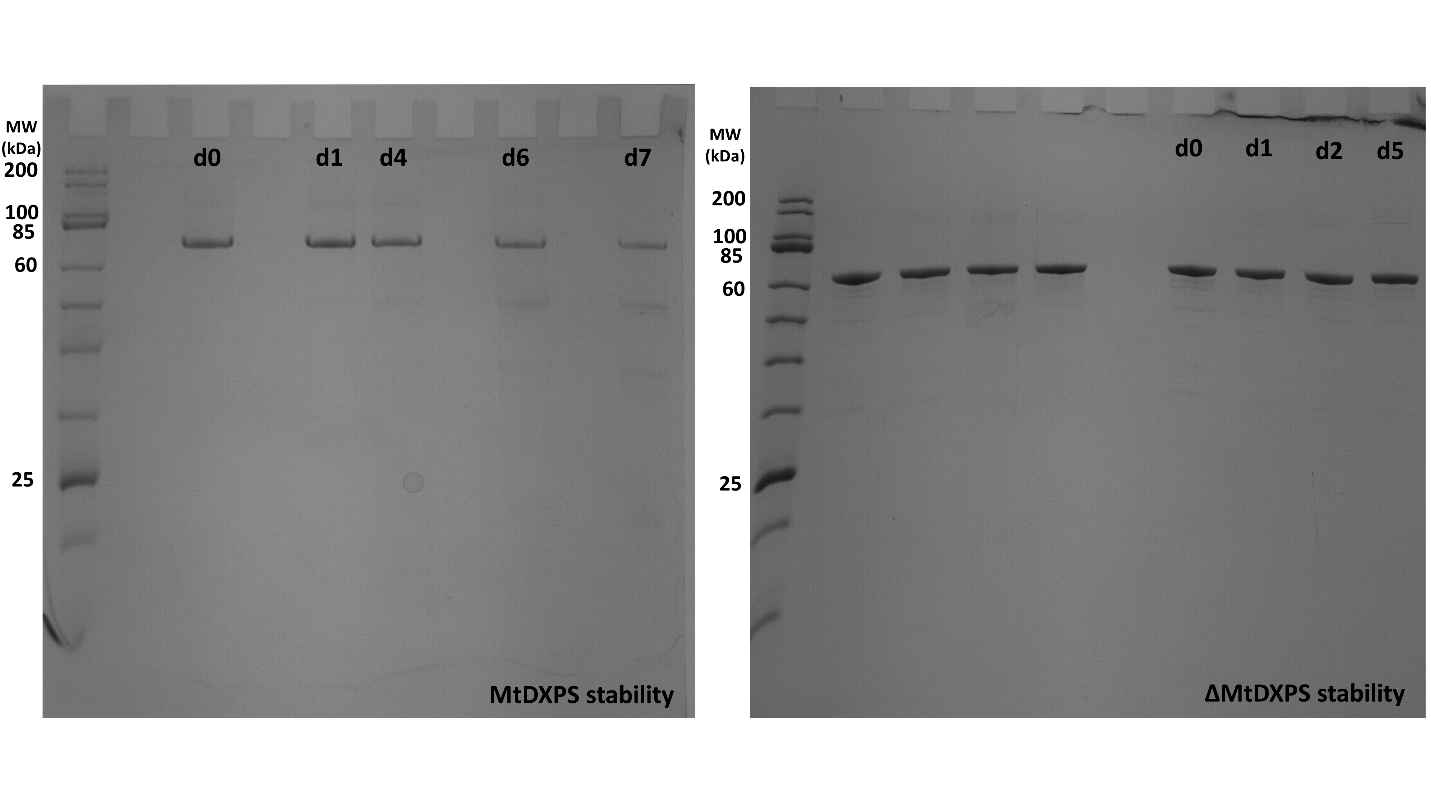


**Figure S3:** SDS-PAGE analysis of MtDXPS (left gel) and ∆MtDXPS (right gel), incubated at RT. The native enzyme is showing bands of degradation products, starting from day one and increasing in intensity over time. Reduction of the main band intensity can be observed from day four on. For the truncated enzyme (lanes 5 to 8), no change of band intensity can be observed up until day five.

### **Enzyme kinetics**

### **
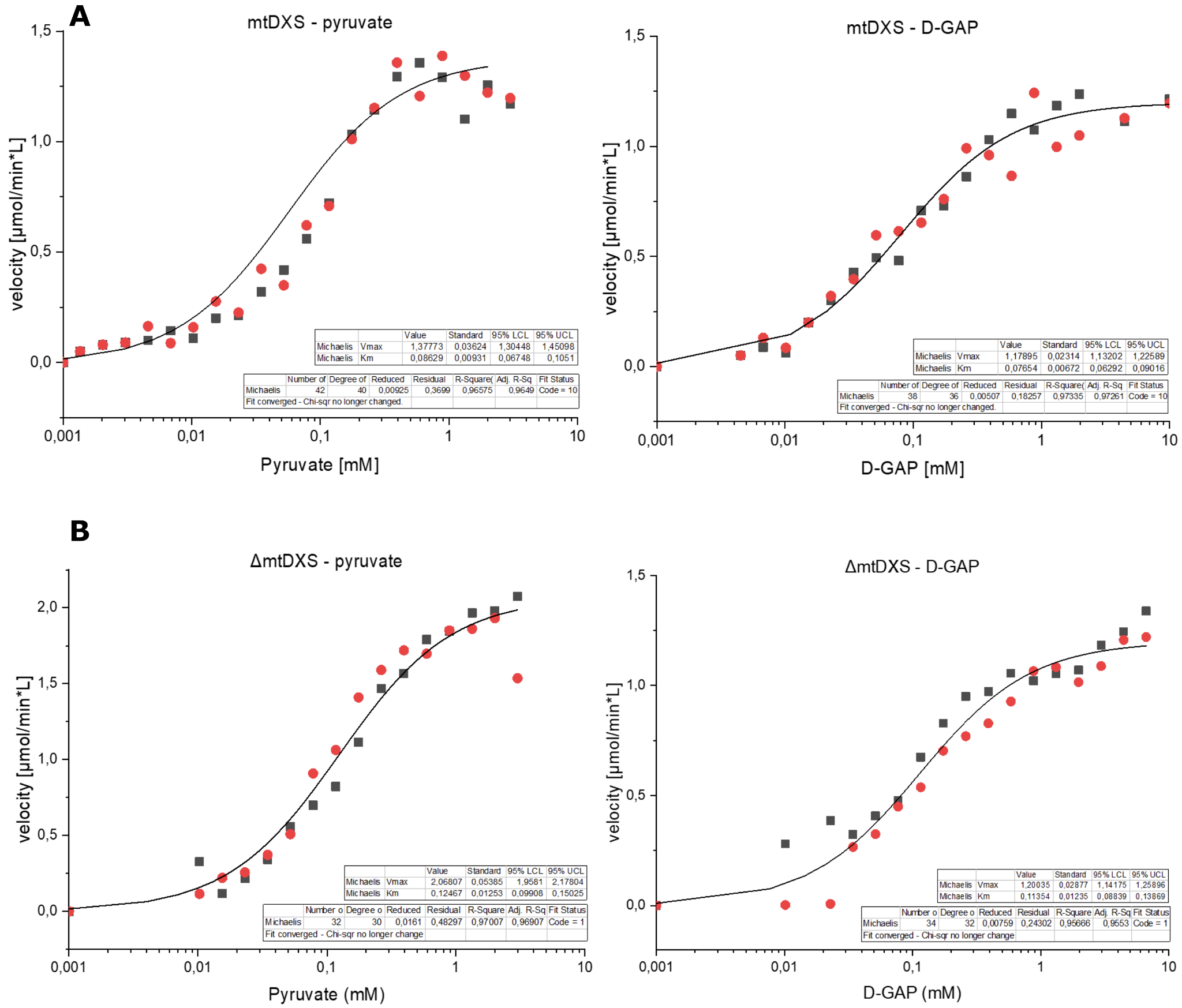
**

**Figure S4:** *K*_m_ determination for the mtDXPS enzymes. **A:** mtDXPS, enzyme concentration used: 5 µmol/L **B:** ∆mtDXPS, enzyme concentration used: 2 µmol/L. Calculation of Kcat was done using the following equation: Vmax / ( [Enz] * 60 ) = Kcat * s^-1

### **MEP pathway enzymes**

***Table S1:*** *Overview of the MEP-pathway enzymes, the corresponding number of published protein crystal structures and their source organisms. The search was conducted in the PDBe archive in February 2021.*

| Enzyme | Number of structures | Species |
| --- | --- | --- |
| DXPS | 5 | *Escherichia coli, Deinococcus radiodurans* |
| IspC | 74 | *Acinetobacter baumannii, E.coli, Moraxella catarrhalis, M. tuberculosis, Plasmodium falciparum, Staphylococcus schleiferi, Thermotoga maritima, Vibrio vulnificus, Yersinia pestis, Yersinia pseudotuberculosis, Zymomonas mobilis* |
| IspD | 43 | *Anaerococcus prevotii, Arabidopsis thaliana, Bacillus subtilis, Burkholderia thailandensis, Campylobacter jejuni, E. coli, Homo sapiens, Listeria monocytogenes, Mycobacterium avium, M. tuberculosis, Neisseria gonorrhoeae, T. maritima, Thermus thermophilus* |
| IspE | 16 | *Aquifex aeolicus, M. tuberculosis, T. thermophilus* |
| IspF | 70 | *A. thaliana, B. subtilis, Burkholderia cenocepacia, Burkholderia pseudomallei, C. jejuni, E. coli, Francisella tularensis, Haemophilus influenza, Mycolicibacterium smegmatis, P. falciparum, Plasmodium vivax, Salmonella enterica, Shewanella oneidensis, Thermus thermophiles, Y. pestis* |
| IspG | 13 | *Aquifex aeolicus, Bacillus anthracis, T. thermophilus* |
| IspH | 12 | *E. coli, P. falciparum* |

###

###

### **Collection and refinement statistics**

### ***Table S2:*** Collection and refinement statistics

| **PDB-ID:** | **7A9H** | **7A9G** |
| --- | --- | --- |
| Wavelength | 1.033 | 1.033 |
| Resolution range | 43.79 - 1.85 (1.916 - 1.85) | 48.5 - 1.9 (1.968 - 1.9) |
| Space group | P 1 | P 1 21 1 |
| Unit cell | 62.987 76.1 79.148 108.956 106.53 107.594 | 62.865 126.17 79.031 90 106.285 90 |
| Total reflections | 165951 (10815) | 175878 (17114) |
| Unique reflections | 93233 (6412) | 92339 (9196) |
| Multiplicity | 1.8 (1.7) | 1.9 (1.9) |
| Completeness (%) | 91.00 (62.53) | 99.39 (99.38) |
| Mean I/sigma(I) | 20.99 (6.54) | 12.40 (2.52) |
| Wilson B-factor | 18.62 | 22.12 |
| R-merge | 0.02334 (0.08525) | 0.04858 (0.3653) |
| R-meas | 0.03298 (0.1205) | 0.0687 (0.5167) |
| R-pim | 0.0233 (0.08508) | 0.04858 (0.3653) |
| CC1/2 | 0.999 (0.979) | 0.997 (0.779) |
| CC* | 1 (0.995) | 0.999 (0.936) |
| Reflections used in refinement | 93233 (6412) | 92339 (9195) |
| Reflections used for R-free | 2100 (144) | 971 (97) |
| R-work | 0.1506 (0.1870) | 0.1538 (0.2398) |
| R-free | 0.1736 (0.2225) | 0.1903 (0.2823) |
| CC(work) | 0.960 (0.904) | 0.956 (0.720) |
| CC(free) | 0.947 (0.916) | 0.958 (0.684) |
| Number of non-hydrogen atoms | 9028 | 8767 |
| macromolecules | 8308 | 8147 |
| ligands | 54 | 220 |
| solvent | 666 | 400 |
| Protein residues | 1091 | 1086 |
| RMS(bonds) | 0.014 | 0.014 |
| RMS(angles) | 1.72 | a1.75 |
| Ramachandran favored (%) | 97.96 | 97.85 |
| Ramachandran allowed (%) | 2.04 | 2.15 |
| Ramachandran outliers (%) | 0.00 | 0.00 |
| Rotamer outliers (%) | 0.70 | 0.48 |
| Clashscore | 2.39 | 2.33 |
| Average B-factor | 20.85 | 24.13 |
| macromolecules | 20.47 | 23.50 |
| ligands | 14.64 | 39.85 |
| solvent | 26.03 | 28.30 |

Statistics for the highest-resolution shell are shown in parentheses.

**Table S3:** Uniprot code and Species name of all sequences from the MSA [1] containing the ESSH motif. Duplicates have not been removed.

| **Uniprot Sequence code** | **Species** |
| --- | --- |
| Q4JVB5\|DXS_CORJK | *Corynebacterium jeikeium* |
| Q6NGV3\|DXS_CORDI | *Corynebacterium diphtheriae* |
| Q8FPI2\|DXS_COREF | *Corynebacterium efficiens* |
| A4QEQ9\|DXS_CORGB | *Corynebacterium glutamicum (strain R)* |
| Q8NPB2\|DXS_CORGL | *Corynebacterium glutamicum* |
| B0RC26\|DXS_CLAMS | *Clavibacter michiganensis subsp. sepedonicus* |
| Q6AFD5\|DXS_LEIXX | *Leifsonia xyli subsp. xyli (strain CTCB07)* |
| B2GJ56\|DXS_KOCRD | *Kocuria rhizophila (strain ATCC 9341 / DSM 348)* |
| A9WRA9\|DXS_RENSM | *Renibacterium salmoninarum* |
| A1R5N7\|DXS_PAEAT | *Paenarthrobacter aurescens* |
| B8HH36\|DXS_PSECP | *Pseudarthrobacter chlorophenolicus* |
| A0JVG9\|DXS_ARTS2 | *Arthrobacter sp. (strain FB24)* |
| Q5YTA2\|DXS_NOCFA | *Nocardia farcinica* |
| C0ZYV9\|DXS_RHOE4 | *Rhodococcus erythropolis* |
| Q0S1H1\|DXS_RHOJR | *Rhodococcus jostii* |
| P0A555\|DXS_MYCBO | *Mycobacterium bovis* |
| C1AFE1\|DXS_MYCBT | *Mycobacterium bovis* |
| A1KM20\|DXS_MYCBP | *Mycobacterium bovis (strain BCG / Pasteur 1173P2)* |
| A5U634\|DXS_MYCTA | *Mycobacterium tuberculosis (strain ATCC 25177 / H37Ra)* |
| A0PT40\|DXS_MYCUA | *Mycobacterium ulcerans (strain Agy99)* |
| A0QIL6\|DXS_MYCA1 | *Mycobacterium avium (strain 104)* |
| B8ZQW9\|DXS_MYCLB | *Mycobacterium leprae (strain Br4923)* |
| Q50000\|DXS_MYCLE | *Mycobacterium leprae (strain TN)* |
| A1UF44\|DXS_MYCSK | *Mycobacterium sp. (strain KMS)* |
| Q1B9W8\|DXS_MYCSS | *Mycobacterium sp. (strain MCS)* |
| A3PYK6\|DXS_MYCSJ | *Mycobacterium sp. (strain JLS)* |
| A1T7Z0\|DXS_MYCVP | *Mycolicibacterium vanbaalenii* |
| A4TCS5\|DXS_MYCGI | *Mycolicibacterium gilvum* |
| A0QW19\|DXS_MYCS2 | *Mycolicibacterium smegmatis* |
| Q73W57\|DXS_MYCPA | *Mycolicibacterium paratuberculosis* |
| B1MCU7\|DXS_MYCA9 | *Mycobacteroides abscessus* |
| P9WNS3\|DXS_MYCTU | *Mycobacterium tuberculosis* |
| P9WNS2\|DXS_MYCTO | *Mycobacterium tuberculosis* |

### **Sequences**

**>sp|P9WNS3|DXS_MYCTU** 1-deoxy-D-xylulose-5-phosphate synthase OS=*Mycobacterium tuberculosis* (strain ATCC 25618 / H37Rv) OX=83332 GN=dxs PE=1 SV=1

MLQQIRGPADLQHLSQAQLRELAAEIREFLIHKVAATGGHLGPNLGVVELTLALHRVFDSPHDPIIFDTGHQAYVHKMLTGRSQDFATLRKKGGLSGYPSRAESEHDWVESSHASAALSYADGLAKAFELTGHRNRHVVAVVGDGALTGGMCWEALNNIAASRRPVIIVVNDNGRSYAPTIGGVADHLATLRLQPAYEQALETGRDLVRAVPLVGGLWFRFLHSVKAGIKDSLSPQLLFTDLGLKYVGPVDGHDERAVEVALRSARRFGAPVIVHVVTRKGMGYPPAEADQAEQMHSTVPIDPATGQATKVAGPGWTATFSDALIGYAQKRRDIVAITAAMPGPTGLTAFGQRFPDRLFDVGIAEQHAMTSAAGLAMGGLHPVVAIYSTFLNRAFDQIMMDVALHKLPVTMVLDRAGITGSDGASHNGMWDLSMLGIVPGIRVAAPRDATRLREELGEALDVDDGPTALRFPKGDVGEDISALERRGGVDVLAAPADGLNHDVLLVAIGAFAPMALAVAKRLHNQGIGVTVIDPRWVLPVSDGVRELAVQHKLLVTLEDNGVNGGAGSAVSAALRRAEIDVPCRDVGLPQEFYEHASRSEVLADLGLTDQDVARRITGWVAALGTGVCASDAIPEHLD

**>Δ*Mt*DXPS** MKHHHHHHPMSDYDIPTTENLYFQGAMGMLQQIRGPADLQHLSQAQLRELAAEIREFLIHKVAATGGHLGPNLGVVELTLALHRVFDSPHDPIIFDTGHQAYVHKMLTGRSQDFATLRKKGGLSGYPSRAESEHDWVESSHASAALSYADGLAKAFELTGHRNRHVVAVVGDGALTGGMCWEALNNIAASRRPVIIVVNDNGRSYAPTIGGVADHLAGGGGGGGPQLLFTDLGLKYVGPVDGHDERAVEVALRSARRFGAPVIVHVVTRKGMGYPPAEADQAEQMHSTVPIDPATGQATKVAGPGWTATFSDALIGYAQKRRDIVAITAAMPGPTGLTAFGQRFPDRLFDVGIAEQHAMTSAAGLAMGGLHPVVAIYSTFLNRAFDQIMMDVALHKLPVTMVLDRAGITGSDGASHNGMWDLSMLGIVPGIRVAAPRDATRLREELGEALDVDDGPTALRFPKGDVGEDISALERRGGVDVLAAPADGLNHDVLLVAIGAFAPMALAVAKRLHNQGIGVTVIDPRWVLPVSDGVRELAVQHKLLVTLEDNGVNGGAGSAVSAALRRAEIDVPCRDVGLPQEFYEHASRSEVLADLGLTDQDVARRITGWVAALGTGVCASDAIPEHLD

1. Gierse, R. M., Reddem, E. R., Alhayek, A., Baitinger, D., Hamid, Z., Jakobi, H., Laber, B., Lange, G., Hirsch, A. K. H., and Groves, M. R. (2021) Identification of a 1-deoxy-D-xylulose-5-phosphate synthase (DXS) mutant with improved crystallographic properties. *Biochem. Biophys. Res. Commun.* **539**, 42–47
